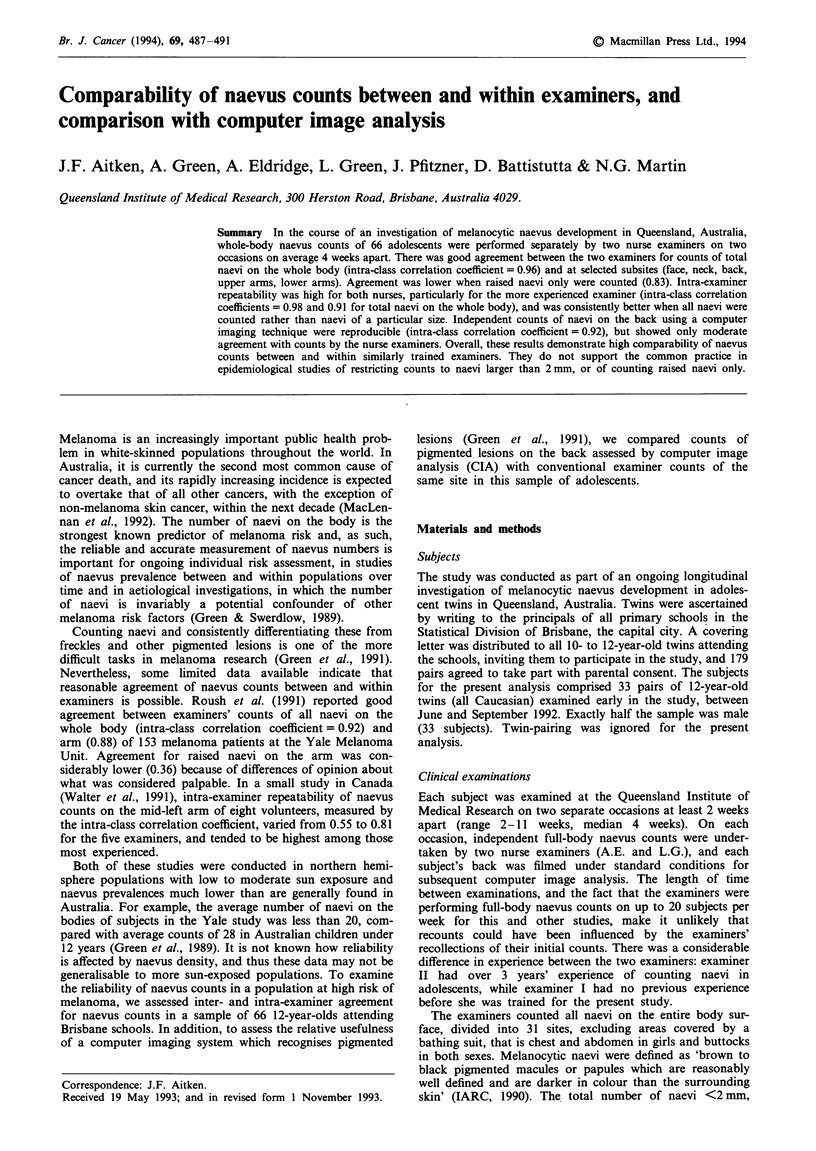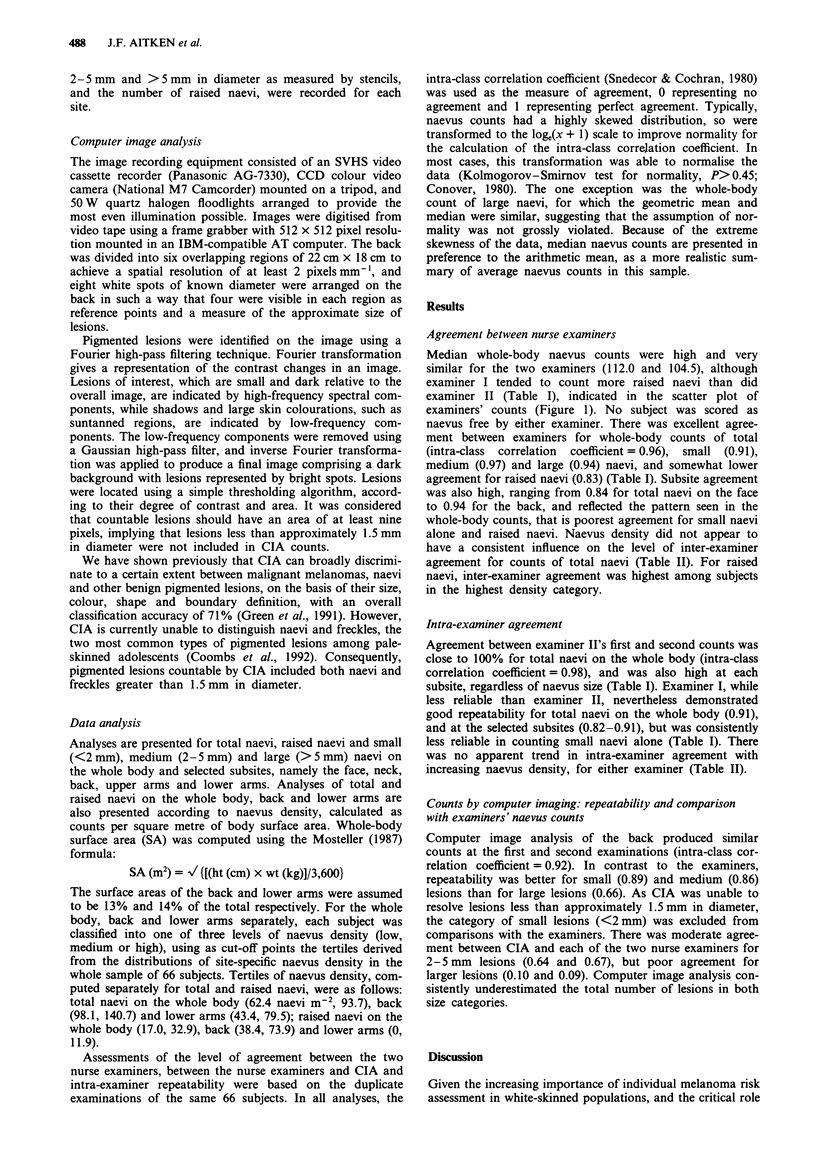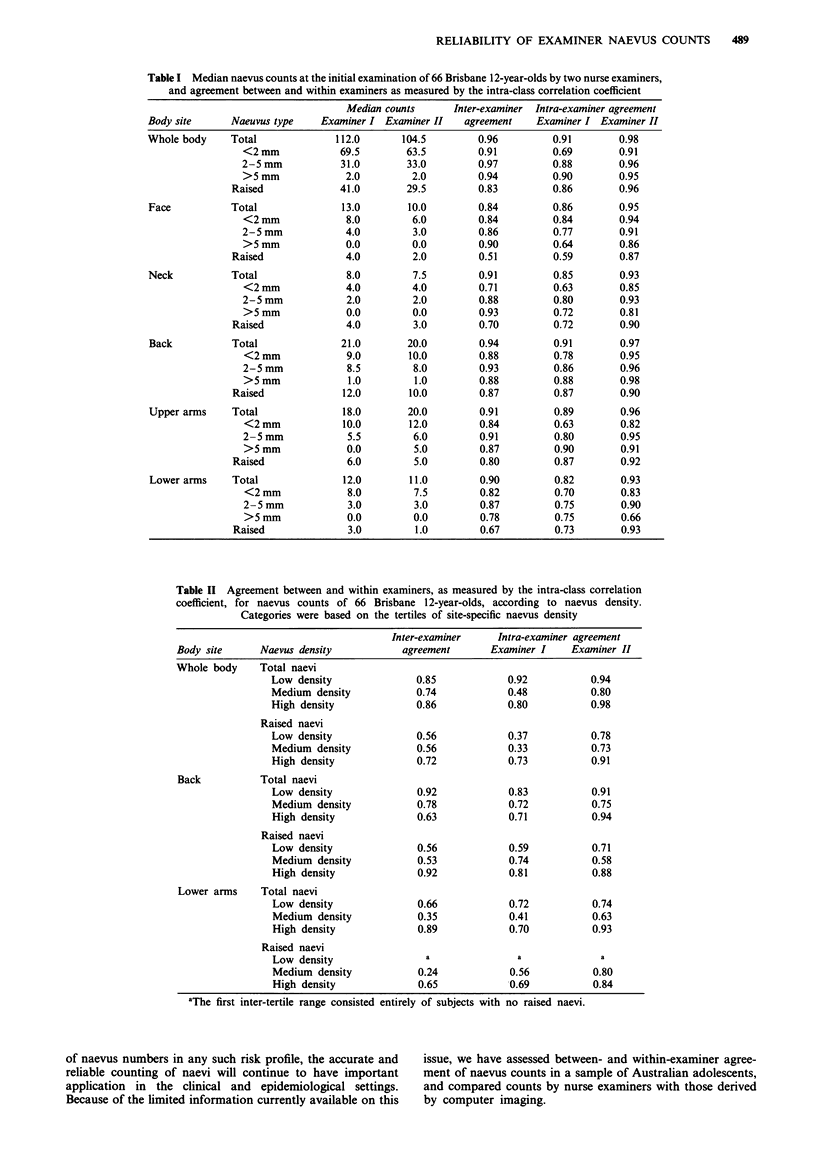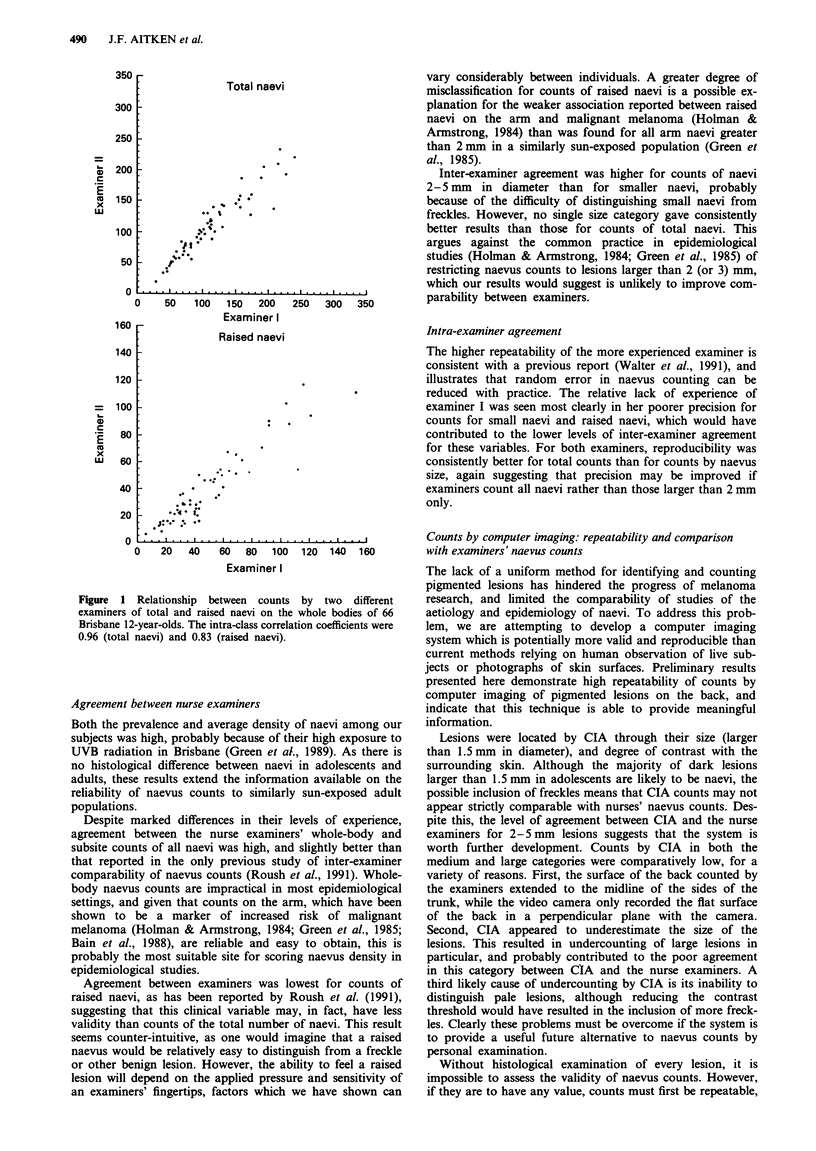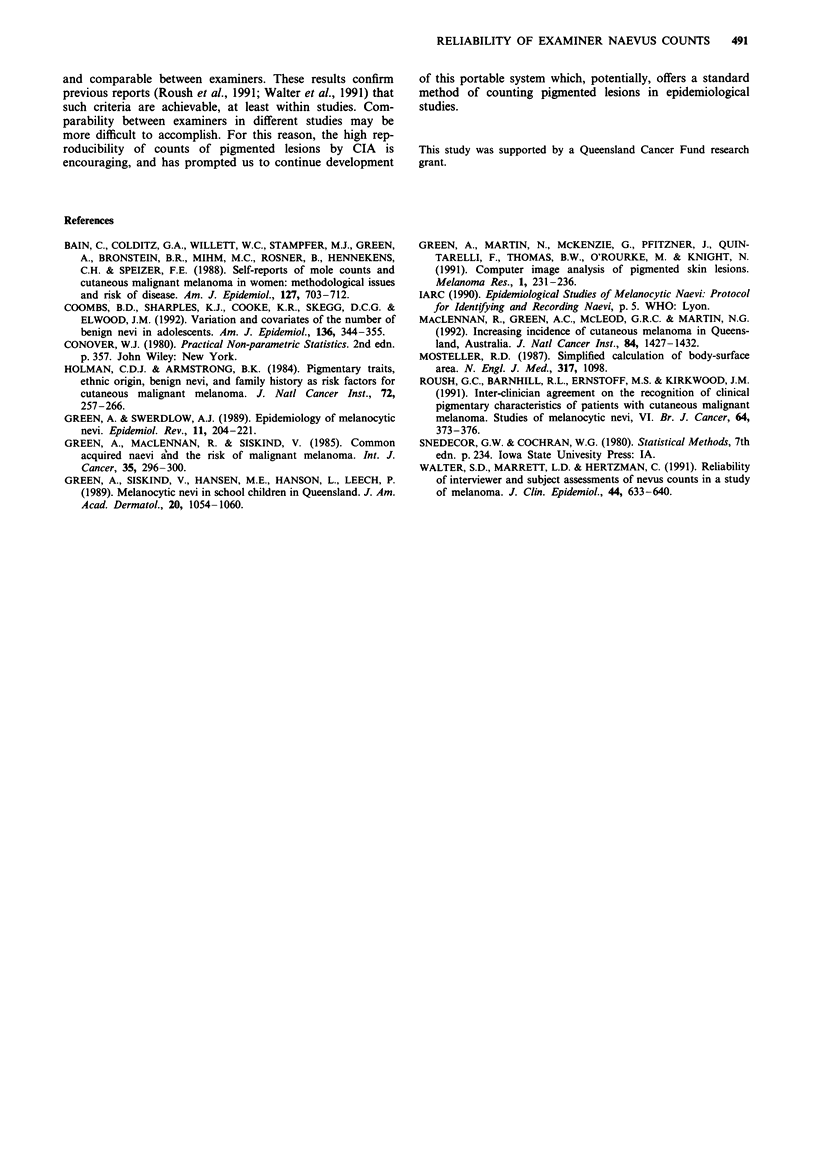# Comparability of naevus counts between and within examiners, and comparison with computer image analysis.

**DOI:** 10.1038/bjc.1994.88

**Published:** 1994-03

**Authors:** J. F. Aitken, A. Green, A. Eldridge, L. Green, J. Pfitzner, D. Battistutta, N. G. Martin

**Affiliations:** Queensland Institute of Medical Research, Brisbane, Australia.

## Abstract

In the course of an investigation of melanocytic naevus development in Queensland, Australia, whole-body naevus counts of 66 adolescents were performed separately by two nurse examiners on two occasions on average 4 weeks apart. There was good agreement between the two examiners for counts of total naevi on the whole body (intra-class correlation coefficient = 0.96) and at selected subsites (face, neck, back, upper arms, lower arms). Agreement was lower when raised naevi only were counted (0.83). Intra-examiner repeatability was high for both nurses, particularly for the more experienced examiner (intra-class correlation coefficients = 0.98 and 0.91 for total naevi on the whole body), and was consistently better when all naevi were counted rather than naevi of a particular size. Independent counts of naevi on the back using a computer imaging technique were reproducible (intra-class correlation coefficient = 0.92), but showed only moderate agreement with counts by the nurse examiners. Overall, these results demonstrate high comparability of naevus counts between and within similarly trained examiners. They do not support the common practice in epidemiological studies of restricting counts to naevi larger than 2 mm, or of counting raised naevi only.


Br. J. Cancer (1994), 69, 487-491

© Macmillan Press Ltd., 1994

### Comparability of naevus counts between and within examiners, and comparison with computer image analysis

J.F. Aitken, A. Green, A. Eldridge, L. Green, J. Pfitzner, D. Battistutta & N.G. Martin

*Queensland Institute of Medical Research, 300 Herston Road, Brisbane, Australia 4029.*

**Summary** In the course of an investigation of melanocytic naevus development in Queensland, Australia, whole-body naevus counts of 66 adolescents were performed separately by two nurse examiners on two occasions on average 4 weeks apart. There was good agreement between the two examiners for counts of total naevi on the whole body (intra-class correlation coefficient = 0.96) and at selected subsites (face, neck, back, upper arms, lower arms). Agreement was lower when raised naevi only were counted (0.83). Intra-examiner repeatability was high for both nurses, particularly for the more experienced examiner (intra-class correlation coefficients = 0.98 and 0.91 for total naevi on the whole body), and was consistently better when all naevi were counted rather than naevi of a particular size. Independent counts of naevi on the back using a computer imaging technique were reproducible (intra-class correlation coefficient = 0.92), but showed only moderate agreement with counts by the nurse examiners. Overall, these results demonstrate high comparability of naevus counts between and within similarly trained examiners. They do not support the common practice in epidemiological studies of restricting counts to naevi larger than 2 mm, or of counting raised naevi only.

Melanoma is an increasingly important public health problem in white-skinned populations throughout the world. In Australia, it is currently the second most common cause of cancer death, and its rapidly increasing incidence is expected to overtake that of all other cancers, with the exception of non-melanoma skin cancer, within the next decade (MacLennan *et al.*, 1992). The number of naevi on the body is the strongest known predictor of melanoma risk and, as such, the reliable and accurate measurement of naevus numbers is important for ongoing individual risk assessment, in studies of naevus prevalence between and within populations over time and in aetiological investigations, in which the number of naevi is invariably a potential confounder of other melanoma risk factors (Green & Swerdlow, 1989).

Counting naevi and consistently differentiating these from freckles and other pigmented lesions is one of the more difficult tasks in melanoma research (Green *et al.*, 1991). Nevertheless, some limited data available indicate that reasonable agreement of naevus counts between and within examiners is possible. Roush *et al.* (1991) reported good agreement between examiners' counts of all naevi on the whole body (intra-class correlation coefficient = 0.92) and arm (0.88) of 153 melanoma patients at the Yale Melanoma Unit. Agreement for raised naevi on the arm was considerably lower (0.36) because of differences of opinion about what was considered palpable. In a small study in Canada (Walter *et al.*, 1991), intra-examiner repeatability of naevus counts on the mid-left arm of eight volunteers, measured by the intra-class correlation coefficient, varied from 0.55 to 0.81 for the five examiners, and tended to be highest among those most experienced.

Both of these studies were conducted in northern hemisphere populations with low to moderate sun exposure and naevus prevalences much lower than are generally found in Australia. For example, the average number of naevi on the bodies of subjects in the Yale study was less than 20, compared with average counts of 28 in Australian children under 12 years (Green *et al.*, 1989). It is not known how reliability is affected by naevus density, and thus these data may not be generalisable to more sun-exposed populations. To examine the reliability of naevus counts in a population at high risk of melanoma, we assessed inter- and intra-examiner agreement for naevus counts in a sample of 66 12-year-olds attending Brisbane schools. In addition, to assess the relative usefulness of a computer imaging system which recognises pigmented lesions (Green *et al.*, 1991), we compared counts of pigmented lesions on the back assessed by computer image analysis (CIA) with conventional examiner counts of the same site in this sample of adolescents.

Correspondence: J.F. Aitken.
Received 19 May 1993; and in revised form 1 November 1993.

### Materials and methods

### *Subjects*

The study was conducted as part of an ongoing longitudinal investigation of melanocytic naevus development in adolescent twins in Queensland, Australia. Twins were ascertained by writing to the principals of all primary schools in the Statistical Division of Brisbane, the capital city. A covering letter was distributed to all 10- to 12-year-old twins attending the schools, inviting them to participate in the study, and 179 pairs agreed to take part with parental consent. The subjects for the present analysis comprised 33 pairs of 12-year-old twins (all Caucasian) examined early in the study, between June and September 1992. Exactly half the sample was male (33 subjects). Twin-pairing was ignored for the present analysis.

### *Clinical examinations*

Each subject was examined at the Queensland Institute of Medical Research on two separate occasions at least 2 weeks apart (range 2–11 weeks, median 4 weeks). On each occasion, independent full-body naevus counts were undertaken by two nurse examiners (A.E. and L.G.), and each subject's back was filmed under standard conditions for subsequent computer image analysis. The length of time between examinations, and the fact that the examiners were performing full-body naevus counts on up to 20 subjects per week for this and other studies, make it unlikely that recounts could have been influenced by the examiners' recollections of their initial counts. There was a considerable difference in experience between the two examiners: examiner II had over 3 years' experience of counting naevi in adolescents, while examiner I had no previous experience before she was trained for the present study.

The examiners counted all naevi on the entire body surface, divided into 31 sites, excluding areas covered by a bathing suit, that is chest and abdomen in girls and buttocks in both sexes. Melanocytic naevi were defined as 'brown to black pigmented macules or papules which are reasonably well defined and are darker in colour than the surrounding skin' (IARC, 1990). The total number of naevi <2 mm,

2–5 mm and >5 mm in diameter as measured by stencils, and the number of raised naevi, were recorded for each site.

*Computer image analysis*

The image recording equipment consisted of an SVHS video cassette recorder (Panasonic AG-7330), CCD colour video camera (National M7 Camcorder) mounted on a tripod, and 50 W quartz halogen floodlights arranged to provide the most even illumination possible. Images were digitised from video tape using a frame grabber with 512 × 512 pixel resolution mounted in an IBM-compatible AT computer. The back was divided into six overlapping regions of 22 cm × 18 cm to achieve a spatial resolution of at least 2 pixels $mm^{-1}$, and eight white spots of known diameter were arranged on the back in such a way that four were visible in each region as reference points and a measure of the approximate size of lesions.

Pigmented lesions were identified on the image using a Fourier high-pass filtering technique. Fourier transformation gives a representation of the contrast changes in an image. Lesions of interest, which are small and dark relative to the overall image, are indicated by high-frequency spectral components, while shadows and large skin colourations, such as suntanned regions, are indicated by low-frequency components. The low-frequency components were removed using a Gaussian high-pass filter, and inverse Fourier transformation was applied to produce a final image comprising a dark background with lesions represented by bright spots. Lesions were located using a simple thresholding algorithm, according to their degree of contrast and area. It was considered that countable lesions should have an area of at least nine pixels, implying that lesions less than approximately 1.5 mm in diameter were not included in CIA counts.

We have shown previously that CIA can broadly discriminate to a certain extent between malignant melanomas, naevi and other benign pigmented lesions, on the basis of their size, colour, shape and boundary definition, with an overall classification accuracy of 71% (Green *et al.*, 1991). However, CIA is currently unable to distinguish naevi and freckles, the two most common types of pigmented lesions among pale-skinned adolescents (Coombs *et al.*, 1992). Consequently, pigmented lesions countable by CIA included both naevi and freckles greater than 1.5 mm in diameter.

*Data analysis*

Analyses are presented for total naevi, raised naevi and small (<2 mm), medium (2–5 mm) and large (>5 mm) naevi on the whole body and selected subsites, namely the face, neck, back, upper arms and lower arms. Analyses of total and raised naevi on the whole body, back and lower arms are also presented according to naevus density, calculated as counts per square metre of body surface area. Whole-body surface area (SA) was computed using the Mosteller (1987) formula:

$$SA\ (m^2) = \sqrt{\{[(ht\ (cm) \times wt\ (kg)]/3,600\}}$$

The surface areas of the back and lower arms were assumed to be 13% and 14% of the total respectively. For the whole body, back and lower arms separately, each subject was classified into one of three levels of naevus density (low, medium or high), using as cut-off points the tertiles derived from the distributions of site-specific naevus density in the whole sample of 66 subjects. Tertiles of naevus density, computed separately for total and raised naevi, were as follows: total naevi on the whole body (62.4 naevi $m^{-2}$, 93.7), back (98.1, 140.7) and lower arms (43.4, 79.5); raised naevi on the whole body (17.0, 32.9), back (38.4, 73.9) and lower arms (0, 11.9).

Assessments of the level of agreement between the two nurse examiners, between the nurse examiners and CIA and intra-examiner repeatability were based on the duplicate examinations of the same 66 subjects. In all analyses, the intra-class correlation coefficient (Snedecor & Cochran, 1980) was used as the measure of agreement, 0 representing no agreement and 1 representing perfect agreement. Typically, naevus counts had a highly skewed distribution, so were transformed to the $\log_e(x + 1)$ scale to improve normality for the calculation of the intra-class correlation coefficient. In most cases, this transformation was able to normalise the data (Kolmogorov–Smirnov test for normality, $P > 0.45$; Conover, 1980). The one exception was the whole-body count of large naevi, for which the geometric mean and median were similar, suggesting that the assumption of normality was not grossly violated. Because of the extreme skewness of the data, median naevus counts are presented in preference to the arithmetic mean, as a more realistic summary of average naevus counts in this sample.

### Results

*Agreement between nurse examiners*

Median whole-body naevus counts were high and very similar for the two examiners (112.0 and 104.5), although examiner I tended to count more raised naevi than did examiner II (Table I), indicated in the scatter plot of examiners' counts (Figure 1). No subject was scored as naevus free by either examiner. There was excellent agreement between examiners for whole-body counts of total (intra-class correlation coefficient = 0.96), small (0.91), medium (0.97) and large (0.94) naevi, and somewhat lower agreement for raised naevi (0.83) (Table I). Subsite agreement was also high, ranging from 0.84 for total naevi on the face to 0.94 for the back, and reflected the pattern seen in the whole-body counts, that is poorest agreement for small naevi alone and raised naevi. Naevus density did not appear to have a consistent influence on the level of inter-examiner agreement for counts of total naevi (Table II). For raised naevi, inter-examiner agreement was highest among subjects in the highest density category.

*Intra-examiner agreement*

Agreement between examiner II's first and second counts was close to 100% for total naevi on the whole body (intra-class correlation coefficient = 0.98), and was also high at each subsite, regardless of naevus size (Table I). Examiner I, while less reliable than examiner II, nevertheless demonstrated good repeatability for total naevi on the whole body (0.91), and at the selected subsites (0.82–0.91), but was consistently less reliable in counting small naevi alone (Table I). There was no apparent trend in intra-examiner agreement with increasing naevus density, for either examiner (Table II).

*Counts by computer imaging: repeatability and comparison with examiners' naevus counts*

Computer image analysis of the back produced similar counts at the first and second examinations (intra-class correlation coefficient = 0.92). In contrast to the examiners, repeatability was better for small (0.89) and medium (0.86) lesions than for large lesions (0.66). As CIA was unable to resolve lesions less than approximately 1.5 mm in diameter, the category of small lesions (<2 mm) was excluded from comparisons with the examiners. There was moderate agreement between CIA and each of the two nurse examiners for 2–5 mm lesions (0.64 and 0.67), but poor agreement for larger lesions (0.10 and 0.09). Computer image analysis consistently underestimated the total number of lesions in both size categories.

### Discussion

Given the increasing importance of individual melanoma risk assessment in white-skinned populations, and the critical role

**Table I** Median naevus counts at the initial examination of 66 Brisbane 12-year-olds by two nurse examiners, and agreement between and within examiners as measured by the intra-class correlation coefficient

| | | *Median counts* | | *Inter-examiner* | *Intra-examiner agreement* | |
|---|---|---|---|---|---|---|
| *Body site* | *Naeuvus type* | *Examiner I* | *Examiner II* | *agreement* | *Examiner I* | *Examiner II* |
| Whole body | Total | 112.0 | 104.5 | 0.96 | 0.91 | 0.98 |
| | <2 mm | 69.5 | 63.5 | 0.91 | 0.69 | 0.91 |
| | 2–5 mm | 31.0 | 33.0 | 0.97 | 0.88 | 0.96 |
| | >5 mm | 2.0 | 2.0 | 0.94 | 0.90 | 0.95 |
| | Raised | 41.0 | 29.5 | 0.83 | 0.86 | 0.96 |
| Face | Total | 13.0 | 10.0 | 0.84 | 0.86 | 0.95 |
| | <2 mm | 8.0 | 6.0 | 0.84 | 0.84 | 0.94 |
| | 2–5 mm | 4.0 | 3.0 | 0.86 | 0.77 | 0.91 |
| | >5 mm | 0.0 | 0.0 | 0.90 | 0.64 | 0.86 |
| | Raised | 4.0 | 2.0 | 0.51 | 0.59 | 0.87 |
| Neck | Total | 8.0 | 7.5 | 0.91 | 0.85 | 0.93 |
| | <2 mm | 4.0 | 4.0 | 0.71 | 0.63 | 0.85 |
| | 2–5 mm | 2.0 | 2.0 | 0.88 | 0.80 | 0.93 |
| | >5 mm | 0.0 | 0.0 | 0.93 | 0.72 | 0.81 |
| | Raised | 4.0 | 3.0 | 0.70 | 0.72 | 0.90 |
| Back | Total | 21.0 | 20.0 | 0.94 | 0.91 | 0.97 |
| | <2 mm | 9.0 | 10.0 | 0.88 | 0.78 | 0.95 |
| | 2–5 mm | 8.5 | 8.0 | 0.93 | 0.86 | 0.96 |
| | >5 mm | 1.0 | 1.0 | 0.88 | 0.88 | 0.98 |
| | Raised | 12.0 | 10.0 | 0.87 | 0.87 | 0.90 |
| Upper arms | Total | 18.0 | 20.0 | 0.91 | 0.89 | 0.96 |
| | <2 mm | 10.0 | 12.0 | 0.84 | 0.63 | 0.82 |
| | 2–5 mm | 5.5 | 6.0 | 0.91 | 0.80 | 0.95 |
| | >5 mm | 0.0 | 5.0 | 0.87 | 0.90 | 0.91 |
| | Raised | 6.0 | 5.0 | 0.80 | 0.87 | 0.92 |
| Lower arms | Total | 12.0 | 11.0 | 0.90 | 0.82 | 0.93 |
| | <2 mm | 8.0 | 7.5 | 0.82 | 0.70 | 0.83 |
| | 2–5 mm | 3.0 | 3.0 | 0.87 | 0.75 | 0.90 |
| | >5 mm | 0.0 | 0.0 | 0.78 | 0.75 | 0.66 |
| | Raised | 3.0 | 1.0 | 0.67 | 0.73 | 0.93 |

**Table II** Agreement between and within examiners, as measured by the intra-class correlation coefficient, for naevus counts of 66 Brisbane 12-year-olds, according to naevus density. Categories were based on the tertiles of site-specific naevus density

| | | *Inter-examiner* | *Intra-examiner agreement* | |
|---|---|---|---|---|
| *Body site* | *Naevus density* | *agreement* | *Examiner I* | *Examiner II* |
| Whole body | Total naevi | | | |
| | Low density | 0.85 | 0.92 | 0.94 |
| | Medium density | 0.74 | 0.48 | 0.80 |
| | High density | 0.86 | 0.80 | 0.98 |
| | Raised naevi | | | |
| | Low density | 0.56 | 0.37 | 0.78 |
| | Medium density | 0.56 | 0.33 | 0.73 |
| | High density | 0.72 | 0.73 | 0.91 |
| Back | Total naevi | | | |
| | Low density | 0.92 | 0.83 | 0.91 |
| | Medium density | 0.78 | 0.72 | 0.75 |
| | High density | 0.63 | 0.71 | 0.94 |
| | Raised naevi | | | |
| | Low density | 0.56 | 0.59 | 0.71 |
| | Medium density | 0.53 | 0.74 | 0.58 |
| | High density | 0.92 | 0.81 | 0.88 |
| Lower arms | Total naevi | | | |
| | Low density | 0.66 | 0.72 | 0.74 |
| | Medium density | 0.35 | 0.41 | 0.63 |
| | High density | 0.89 | 0.70 | 0.93 |
| | Raised naevi | | | |
| | Low density | [a] | [a] | [a] |
| | Medium density | 0.24 | 0.56 | 0.80 |
| | High density | 0.65 | 0.69 | 0.84 |

[a]The first inter-tertile range consisted entirely of subjects with no raised naevi.

of naevus numbers in any such risk profile, the accurate and reliable counting of naevi will continue to have important application in the clinical and epidemiological settings. Because of the limited information currently available on this issue, we have assessed between- and within-examiner agreement of naevus counts in a sample of Australian adolescents, and compared counts by nurse examiners with those derived by computer imaging.

**Figure 1** Relationship between counts by two different examiners of total and raised naevi on the whole bodies of 66 Brisbane 12-year-olds. The intra-class correlation coefficients were 0.96 (total naevi) and 0.83 (raised naevi).

*Agreement between nurse examiners*

Both the prevalence and average density of naevi among our subjects was high, probably because of their high exposure to UVB radiation in Brisbane (Green *et al.*, 1989). As there is no histological difference between naevi in adolescents and adults, these results extend the information available on the reliability of naevus counts to similarly sun-exposed adult populations.

Despite marked differences in their levels of experience, agreement between the nurse examiners' whole-body and subsite counts of all naevi was high, and slightly better than that reported in the only previous study of inter-examiner comparability of naevus counts (Roush *et al.*, 1991). Whole-body naevus counts are impractical in most epidemiological settings, and given that counts on the arm, which have been shown to be a marker of increased risk of malignant melanoma (Holman & Armstrong, 1984; Green *et al.*, 1985; Bain *et al.*, 1988), are reliable and easy to obtain, this is probably the most suitable site for scoring naevus density in epidemiological studies.

Agreement between examiners was lowest for counts of raised naevi, as has been reported by Roush *et al.* (1991), suggesting that this clinical variable may, in fact, have less validity than counts of the total number of naevi. This result seems counter-intuitive, as one would imagine that a raised naevus would be relatively easy to distinguish from a freckle or other benign lesion. However, the ability to feel a raised lesion will depend on the applied pressure and sensitivity of an examiners' fingertips, factors which we have shown can vary considerably between individuals. A greater degree of misclassification for counts of raised naevi is a possible explanation for the weaker association reported between raised naevi on the arm and malignant melanoma (Holman & Armstrong, 1984) than was found for all arm naevi greater than 2 mm in a similarly sun-exposed population (Green *et al.*, 1985).

Inter-examiner agreement was higher for counts of naevi 2–5 mm in diameter than for smaller naevi, probably because of the difficulty of distinguishing small naevi from freckles. However, no single size category gave consistently better results than those for counts of total naevi. This argues against the common practice in epidemiological studies (Holman & Armstrong, 1984; Green *et al.*, 1985) of restricting naevus counts to lesions larger than 2 (or 3) mm, which our results would suggest is unlikely to improve comparability between examiners.

*Intra-examiner agreement*

The higher repeatability of the more experienced examiner is consistent with a previous report (Walter *et al.*, 1991), and illustrates that random error in naevus counting can be reduced with practice. The relative lack of experience of examiner I was seen most clearly in her poorer precision for counts for small naevi and raised naevi, which would have contributed to the lower levels of inter-examiner agreement for these variables. For both examiners, reproducibility was consistently better for total counts than for counts by naevus size, again suggesting that precision may be improved if examiners count all naevi rather than those larger than 2 mm only.

*Counts by computer imaging: repeatability and comparison with examiners' naevus counts*

The lack of a uniform method for identifying and counting pigmented lesions has hindered the progress of melanoma research, and limited the comparability of studies of the aetiology and epidemiology of naevi. To address this problem, we are attempting to develop a computer imaging system which is potentially more valid and reproducible than current methods relying on human observation of live subjects or photographs of skin surfaces. Preliminary results presented here demonstrate high repeatability of counts by computer imaging of pigmented lesions on the back, and indicate that this technique is able to provide meaningful information.

Lesions were located by CIA through their size (larger than 1.5 mm in diameter), and degree of contrast with the surrounding skin. Although the majority of dark lesions larger than 1.5 mm in adolescents are likely to be naevi, the possible inclusion of freckles means that CIA counts may not appear strictly comparable with nurses' naevus counts. Despite this, the level of agreement between CIA and the nurse examiners for 2–5 mm lesions suggests that the system is worth further development. Counts by CIA in both the medium and large categories were comparatively low, for a variety of reasons. First, the surface of the back counted by the examiners extended to the midline of the sides of the trunk, while the video camera only recorded the flat surface of the back in a perpendicular plane with the camera. Second, CIA appeared to underestimate the size of the lesions. This resulted in undercounting of large lesions in particular, and probably contributed to the poor agreement in this category between CIA and the nurse examiners. A third likely cause of undercounting by CIA is its inability to distinguish pale lesions, although reducing the contrast threshold would have resulted in the inclusion of more freckles. Clearly these problems must be overcome if the system is to provide a useful future alternative to naevus counts by personal examination.

Without histological examination of every lesion, it is impossible to assess the validity of naevus counts. However, if they are to have any value, counts must first be repeatable, and comparable between examiners. These results confirm previous reports (Roush *et al.*, 1991; Walter *et al.*, 1991) that such criteria are achievable, at least within studies. Comparability between examiners in different studies may be more difficult to accomplish. For this reason, the high reproducibility of counts of pigmented lesions by CIA is encouraging, and has prompted us to continue development of this portable system which, potentially, offers a standard method of counting pigmented lesions in epidemiological studies.

This study was supported by a Queensland Cancer Fund research grant.

### References

BAIN, C., COLDITZ, G.A., WILLETT, W.C., STAMPFER, M.J., GREEN, A., BRONSTEIN, B.R., MIHM, M.C., ROSNER, B., HENNEKENS, C.H. & SPEIZER, F.E. (1988). Self-reports of mole counts and cutaneous malignant melanoma in women: methodological issues and risk of disease. *Am. J. Epidemiol.*, **127**, 703–712.

COOMBS, B.D., SHARPLES, K.J., COOKE, K.R., SKEGG, D.C.G. & ELWOOD, J.M. (1992). Variation and covariates of the number of benign nevi in adolescents. *Am. J. Epidemiol.*, **136**, 344–355.

CONOVER, W.J. (1980). *Practical Non-parametric Statistics*. 2nd edn. p. 357. John Wiley: New York.

HOLMAN, C.D.J. & ARMSTRONG, B.K. (1984). Pigmentary traits, ethnic origin, benign nevi, and family history as risk factors for cutaneous malignant melanoma. *J. Natl Cancer Inst.*, **72**, 257–266.

GREEN, A. & SWERDLOW, A.J. (1989). Epidemiology of melanocytic nevi. *Epidemiol. Rev.*, **11**, 204–221.

GREEN, A., MACLENNAN, R. & SISKIND, V. (1985). Common acquired naevi and the risk of malignant melanoma. *Int. J. Cancer*, **35**, 296–300.

GREEN, A., SISKIND, V., HANSEN, M.E., HANSON, L., LEECH, P. (1989). Melanocytic nevi in school children in Queensland. *J. Am. Acad. Dermatol.*, **20**, 1054–1060.

GREEN, A., MARTIN, N., MCKENZIE, G., PFITZNER, J., QUINTARELLI, F., THOMAS, B.W., O'ROURKE, M. & KNIGHT, N. (1991). Computer image analysis of pigmented skin lesions. *Melanoma Res.*, **1**, 231–236.

IARC (1990). *Epidemiological Studies of Melanocytic Naevi: Protocol for Identifying and Recording Naevi*, p. 5. WHO: Lyon.

MACLENNAN, R., GREEN, A.C., MCLEOD, G.R.C. & MARTIN, N.G. (1992). Increasing incidence of cutaneous melanoma in Queensland, Australia. *J. Natl Cancer Inst.*, **84**, 1427–1432.

MOSTELLER, R.D. (1987). Simplified calculation of body-surface area. *N. Engl. J. Med.*, **317**, 1098.

ROUSH, G.C., BARNHILL, R.L., ERNSTOFF, M.S. & KIRKWOOD, J.M. (1991). Inter-clinician agreement on the recognition of clinical pigmentary characteristics of patients with cutaneous malignant melanoma. Studies of melanocytic nevi, VI. *Br. J. Cancer*, **64**, 373–376.

SNEDECOR, G.W. & COCHRAN, W.G. (1980). *Statistical Methods*, 7th edn. p. 234. Iowa State Univesity Press: IA.

WALTER, S.D., MARRETT, L.D. & HERTZMAN, C. (1991). Reliability of interviewer and subject assessments of nevus counts in a study of melanoma. *J. Clin. Epidemiol.*, **44**, 633–640.